# SIGNOR 3.0, the SIGnaling network open resource 3.0: 2022 update

**DOI:** 10.1093/nar/gkac883

**Published:** 2022-10-16

**Authors:** Prisca Lo Surdo, Marta Iannuccelli, Silvia Contino, Luisa Castagnoli, Luana Licata, Gianni Cesareni, Livia Perfetto

**Affiliations:** Fondazione Human Technopole, Milan 20157, Italy; Department of Biology, University of Rome ‘Tor Vergata’, Rome 00133, Italy; Department of Biology, University of Rome ‘Tor Vergata’, Rome 00133, Italy; Department of Biology, University of Rome ‘Tor Vergata’, Rome 00133, Italy; Fondazione Human Technopole, Milan 20157, Italy; Department of Biology, University of Rome ‘Tor Vergata’, Rome 00133, Italy; Fondazione Human Technopole, Milan 20157, Italy

## Abstract

The SIGnaling Network Open Resource (SIGNOR 3.0, https://signor.uniroma2.it) is a public repository that captures causal information and represents it according to an ‘activity-flow’ model. SIGNOR provides freely-accessible static maps of causal interactions that can be tailored, pruned and refined to build dynamic and predictive models. Each signaling relationship is annotated with an effect (up/down-regulation) and with the mechanism (e.g. binding, phosphorylation, transcriptional activation, etc.) causing the regulation of the target entity. Since its latest release, SIGNOR has undergone a significant upgrade including: (i) a new website that offers an improved user experience and novel advanced search and graph tools; (ii) a significant content growth adding up to a total of approx. 33,000 manually-annotated causal relationships between more than 8900 biological entities; (iii) an increase in the number of manually annotated pathways, currently including pathways deregulated by SARS-CoV-2 infection or involved in neurodevelopment synaptic transmission and metabolism, among others; (iv) additional features such as new model to represent metabolic reactions and a new confidence score assigned to each interaction.

## INTRODUCTION

Cell physiology is governed by a complex mesh of interactions between different biological entities: proteins, RNA, metabolites etc. Perturbation of these interaction networks often causes diseases (1). Some of these interactions are physical and lead to the formation of molecular complexes, organelles and, more in general, dictate cell architecture. This interaction type is symmetrical as it has no directionality. Many functional interactions, on the other hand, are directional as the activity of an upstream entity leads to the activation (positive effect) or to the inhibition (negative effect) of the downstream one (2). These interactions are causal and may or may not involve physical contact. The former and the latter form of networks can be represented by undirected and signed-directed graphs, respectively. Several high and low-throughput approaches have been developed to obtain experimental information on the protein interactions that form the physical interaction network (3,4). This information has been captured by expert curators and organized in a computer readable format by a number or resources (5–8). Although some physical interactions have consequences on the activity of one of the partners, the physical and the causal networks are largely complementary.

Experimental evidence on causal interactions is sparse and, while the vast majority of proteins in the human proteome have at least one identified physical partner, for many proteins little or no experimental detail exists about the impact of modulating their activities on other protein activity in the proteome.

Over recent years, we and others have started annotating causal information in public resources (9,10). SIGNOR is presently the database that has manually captured the highest number of causal interactions, which are offered open access and are fully downloadable in a standardized format (11,12). This effort has grown over the years and now the dataset that can be downloaded from the SIGNOR website covers ∼33% of the reference proteome (UP000005640, reviewed dataset, UniProt release 2022_03).

We report in this update the increase in proteome coverage. In addition, we present a new website that we have developed to improve user experience and to introduce new graph and search tools. Finally, we describe a new approach to assign to each interaction a score that estimates the trust that the community places on its biological relevance.

## RESULTS

### Significant content increase

**Figure 1.**
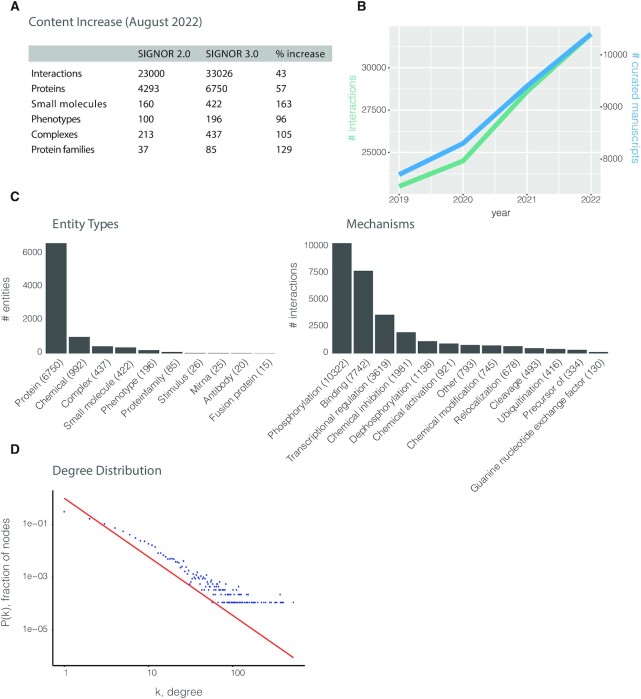
SIGNOR 3.0, content increase. (**A**) Comparison of the causal information captured in SIGNOR 3.0 and 2.0. (**B**) Kinetic of content growth since the publication of SIGNOR 2.0. The green and blue lines represent the growth in the number of annotated interactions and curated manuscripts respectively. (**C**) Histograms showing the number of different ‘Entity types’ (left panel) and the number of interactions linked to different ‘Mechanisms’ (right panel), as annotated in SIGNOR. (**D**) Degree distribution of SIGNOR entities. Scatterplot displaying the degree distribution in the SIGNOR network, with the fitted power law (red).

After the publication of SIGNOR 2.0 (10) the SIGNOR curation team has continued the effort aimed at capturing causal information between biological entities. This work resulted in a significant increase of the information content of the resource. Close to 10,000 new interactions were added to the database by curating over 2600 manuscripts (Figure 1A, B). The vast majority of the interactions annotated in SIGNOR are between proteins. Additional molecular types or biological entities are, however, also considered: complexes, protein families, chemicals, small molecules, phenotypes, stimuli and others (Figure 1C). Particularly significant is the increase in proteome coverage. Approximately 33% of the human proteome (6750 proteins) is now integrated in the SIGNOR network. Each interaction is annotated with the molecular mechanism (e.g. ubiquitination, binding) causing the effect, positive (activation or up-regulation) or negative (inhibition or down-regulation), on the target entity (Figure 1C). Phosphorylation is by far the most frequent annotation in SIGNOR. This places SIGNOR among the main databases for annotation of phosphorylation reactions where the kinase promoting the modification, the modified residue and the effect (activation-inhibition) on the target are known. Some effort was also put on increasing the coverage of transcriptional regulation. Transcriptional regulation validated by evidence of direct interactions between transcription factors and target genes are annotated as direct. As the SIGNOR model also allows annotating indirect interactions, a few indirect transcriptional regulation relationships are captured and clearly labeled as ‘indirect’. No inferred interaction based on high-throughput experiments is considered. This somewhat decreases the coverage in the interest of accuracy and biological relevance.

The degree distribution of the SIGNOR graph, as that of many biological interaction networks, follows a power law with few nodes connected by many interactions while most nodes are connected by only one or few edges (Figure 1D). A novelty of SIGNOR 3.0 is the adoption of a model that permits the integration of metabolic reactions into a causal network (13). This now allows one to explore the crosstalk between signaling and metabolism in a single connected network.

**Figure 2.**
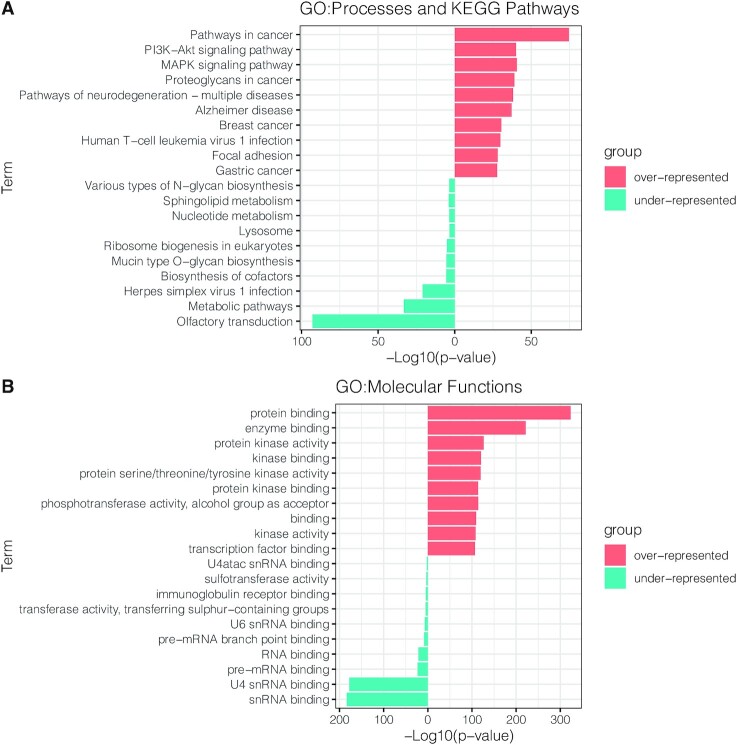
GO and KEGG terms under- and over-represented in the SIGNOR dataset. The two graphs list the GO:Biological Processes and KEGG Pathways (**A**) and GO:Molecular Function terms (**B**) significantly enriched in the human proteins present in the SIGNOR 3.0. Only the top 10 significantly (Bonferroni-adjusted *P*-value < 0.025) over-represented (red) and under-represented (blue) terms are shown. Analyses were carried out by using the gProfiler2 software (32), using the entire Human Proteome as a background.

Although SIGNOR is a generalist database that aims at capturing causal information without concentrating on specific biological domains, we have also undertaken some focused curation campaigns (cancer, COVID, neuropsychiatric disorders, phosphorylation etc.). The bar chart in Figure 2 displays the GO and the KEGG annotations that are over- or under-represented in the proteins in the database. Proteins that are annotated with terms related to phosphorylation, cancer and neurological diseases are over-represented whereas metabolic enzymes are still under-represented. RNA binding proteins are also under-represented and will need more attention in the future.

### A new web interface

#### Appearance and layout

**Figure 3.**
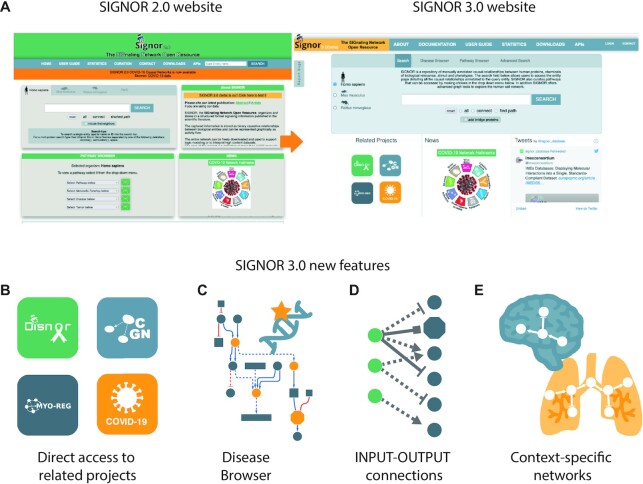
SIGNOR 3.0, new web resource and functionalities. (**A**) The SIGNOR 2.0 and 3.0 home pages. (**B**) SIGNOR 3.0 provides direct access to related projects: DisNor (14), CancerGeneNet (15), Myo-REG (16) and the COVID-19 (17). (**C**) The disease browser builds causal network connecting genes that have been found to be mutated in patients affected by a specific disease (GDA, nodes in yellow), as provided by the DisGeNET (20), IntOGen (21) and Cancer Gene Census (22) resources. (**D**) Newly developed ‘advanced methods’ allow the user to browse for direct connections linking custom lists of input and output proteins. (**E**) Newly developed ‘advanced methods’ allow the user to filter interactions annotated to a given tissue, cell type or system.

While the SIGNOR database structure remains unchanged, we largely redesigned the SIGNOR 3.0 web site, according to users’ comments and suggestions. One of the aspects we focused on, while developing the new version of the web interface, was the design of a homepage that would improve usability and user experience (Figure 3A). To this end, we reconsidered the elements that should be immediately available to the user and how their layout would affect accessibility. The updated color palette preserves some of the original elements of the SIGNOR color scheme while also adding fresh ones to highlight important features. The new design calls attention to the various search options by centering on one main search box and improves user-friendliness through a tab system that reduces confusion among various functions, by focusing on a selected few at a time.

#### Functionality

The most important updates of the newly redesigned resource are within the scope of functionality. These include 1) the direct access to SIGNOR-related projects (Figure 3B), namely, DisNor (14), CancerGeneNet (15), Myo-REG (16) and the COVID-19 (17); 2) novel search and filtering options, organized as tabs in the search box. Briefly, the tabs ‘Search’, ‘Disease Browser’, ‘Pathway Browser’ and ‘Advanced methods’ give access to search and graph tools to explore the SIGNOR dataset (Figure 3A, Supplementary Figure S1A). The ‘search’ and the ‘pathway browser’ have undergone cosmetic changes and have been modified to minimize crowding and improve usability. In addition, new functionalities, such as the ‘Disease Browser’ and the ‘Advanced methods’ were implemented to improve filtering options and accessibility to the resource.

#### Searching SIGNOR 3.0, search tab

The ‘search’ tab gives access to the default search tool. Users can enter in a text field one or more entity names or identifiers. If a single entity name is entered, the tool redirects the user to an entity page showing functional details and all the causal interactions of the query entity. If more than one entity is entered in the search field the tool returns (i) a network formed by all the interactions of the query proteins, if the *‘all’* radio button is selected; (ii) a network showing all the direct interactions between the query proteins, when the ‘*connect*’ radio button is checked; (iii) a network connecting the query entities that are separated by two edges or fewer when both the *‘connect’* and the *‘add bridge proteins* ’ are checked. Finally, when two entities are entered and the user selects the *‘find path*’ radio button the tool searches and displays all the directional graph paths that connect the two query proteins.

#### Searching SIGNOR 3.0, pathway browser tab

SIGNOR—unlike pathway-centric resources like KEGG and Reactome (18,19)—is a *‘causal interaction database*’, where literature-captured relationships are merged to form a large, intricate and connected interactome. However, part of the SIGNOR curation effort is directed at annotating sub-networks (pathways) underlying the cellular response to specific environmental stimuli. In this model the cellular pathways in SIGNOR are embedded in the global interactome, thereby allowing for exploration of pathway cross-talk. The Pathway Browser tab grants access to 114 manually curated pathways that are organized in five categories: *Pathway, Metabolic Pathway, Disease, Tumor* and *Module*. *Modules* are defined as smaller networks that underlie commonly perturbed signal transduction pathways.

### New search options

Two new tools that are offered by SIGNOR 3.0 can be accessed via the ‘Disease Browser’ and ‘Advanced methods’ tabs.

#### Disease Browser

The Disease Browser tool allows the user to access three resources compiling disease associated gene lists (GDAs): DisGeNET, IntOGen and Cancer Gene Census (20–22). Briefly, the tool takes as input a user selected disease and searches the SIGNOR dataset for relationships causally connecting the genes that are associated to the query disease by the three resources (Figure 3C). The output is a disease specific graph that shows the causal connections between disease genes. The user is also offered the possibility to choose between three graph algorithms: *all*, *connect* and *add bridge proteins* as described in the ‘Searching SIGNOR 3.0’ paragraph (Supplementary Figure S2B) (14). At the time of writing, the number of diseases that can be accessed by this tool is over 4200.

#### Advanced methods

SIGNOR 3.0 also offers new ‘Advanced methods’ designed to ask more complex queries and to filter and refine search results. The tool allows one to search for entries where a query protein or a list of query proteins play the roles of regulators or targets (Figure 3D). Additionally, a series of drop-down menus permit to filter the results and show only relations that are annotated with metadata related to (i) the experimental system used to demonstrate the interaction (Organism, cell type, tissue etc.). This information is inferred from contextual evidence from the supporting publication and manually captured from our curators (Figure 3E); (ii) the mechanism underlying the causal relationship (phosphorylation, transcriptional regulation etc.) or the (iii) ‘effect’ (up or down-regulation). As 90% of the SIGNOR interactions are annotated with the molecular mechanism endorsing the causal link (Figure 1C) and approximately one third with the experimental system that was used to demonstrate the interaction (cell type, tissue etc.) (Supplementary Figure S4) this new tool makes now possible to filter the SIGNOR interactome to retain, for instance, only interactions annotated to the ‘Nervous system’ or to retrieve all the phosphorylations events captured in SIGNOR, thus fully exploiting the metadata linked to each interaction.

The ‘Advanced methods’ page also includes a tool that infers the regulatory crosstalk between one or more proteins and a curated pathway. This tool takes as input a list of proteins and one of the manually curated pathways and searches for paths (of two or fewer steps) connecting the input entities to the selected pathway. If such paths exist a visual representation of the result is given in output (Supplementary Figure S3).

### New SIGNOR 3.0 score

Since the latest release, the procedure to assign a confidence score to each interaction was updated as detailed in Supplementary materials (Supplementary Figure S5). Briefly, a principal component regression approach (23) is used to optimize a model that integrates a number of molecular features (e.g. the amount of experimental evidence in SIGNOR and score values extracted from the STRING database (24)) to predict whether a relationship between an entity pair is part of a gold standard (i.e. part of molecular pathways in the resource). A detailed description can be downloaded from the documentation section of the web page (https://signor.uniroma2.it/documentation/).

## DISCUSSION

SIGNOR is a public repository of signaling information, fully compliant with the FAIR principles, which grant for data Findability, Accessibility, Interoperability and Reusability (25).

Version 3.0 presents significant advancements in the value of the SIGNOR resource. To improve user experience and data accessibility, we redesigned the web resource and added new search and analysis tools. In addition, we also expanded the programmatic access to the resource, by implementing new APIs that are described in the corresponding section and that can be accessed from the Website homepage (https://signor.uniroma2.it/APIs.php). In addition, SIGNOR data is also regularly released via The Network Data Exchange (NDEx), an open-source collection of biological networks, conceived for manipulation, integration and re-use (26). As a fourth way to access the SIGNOR dataset, we recently developed a Cytoscape application (27). SIGNORApp was conceived to functionally mirror the main search types of SIGNOR (e.g. the single and multiple entities search, pathway browser, etc.). For each query, the SIGNORApp generates networks in the form of signed, directed graphs that can be displayed within the framework of the Cytoscape software platform. This enables users to directly apply Cytoscape-compatible tools for analysis and visualization of the SIGNOR dataset. The application is available for Cytoscape 3 (versions 3.8 and higher) and can be freely downloaded from https://apps.cytoscape.org/apps/signorapp.

A steady curation effort has led to a significant increase of the coverage of the human proteome. To date, 33% of it is integrated in the causal interactome, whereas the number of interactions and pathways captured grew by 45% and 130%, respectively.

Complete capture of published causal information remains the main goal of the SIGNOR project. SIGNOR is presently the primary causal interaction database with the highest coverage in the non-commercial domain (11,12). Despite this, we are still far from an exhaustive description of all the causal links in the human proteome. Indeed, for ∼70% of the proteins we still miss information on causal links that would permit us to infer how the activity of upstream proteins influence the state of downstream ones. Capturing this missing information is our mission over the forthcoming years. To get closer to this milestone we plan to triage and re-curate text mining-derived datasets, such as the ExTRI dataset of Transcription Factors-Target genes (28). In addition, we also plan to prioritize curation of proteins and pathways that are under-represented in our resource (Figure 2A).

It is important to stress that all the interactions annotated by SIGNOR curators are mapped to the human proteome and displayed as a single network that describes an abstract cell where all the proteins are expressed and active. The functional interactome that is relevant for the physiology of a specific cell, on the other hand, depends on the cell molecular context and on the level of gene expression of the different genes in the specific cell type. Recent advances in methods that allow to reveal the transcription profile of single cells now permit tuning the interactome of this naive cell to the molecular profile of the different cell types (29). Producing such cell specific interactomes is an important goal that should be achievable reasonably soon.

Finally, although proteins are the main players in the causal interactome, gene expression and signaling are also regulated by other molecular entities. In the new millennium non-coding RNAs have progressively taken a main stage in the gene expression scenery (30,31). The SIGNOR model allows to consider regulation of gene expression mediated by non-coding RNAs. Presently, this area is poorly covered in the SIGNOR interactome. We plan to define a representation model and include these types of regulatory events.

## DATA AVAILABILITY

SIGNOR 3.0 interaction data are available and freely downloadable at: https://signor.uniroma2.it/downloads.php.

Furthermore, programmatic access is available through the APIs section at https://signor.uniroma2.it/APIs.php.

All described database features are accessible via the main SIGNOR 3.0 website: https://signor.uniroma2.it/.

SIGNORApp can be freely downloaded from https://apps.cytoscape.org/apps/signorapp.

## Supplementary Material

gkac883_Supplemental_File
