# Supplementary material for: SIGNOR 3.0, the SIGnaling network open resource 3.0: 2022 update
Source: Nucleic Acids Res. 2022 Oct 16;51(D1):D631–7. doi: 10.1093/nar/gkac883 (PMC9825604; doi:10.1093/nar/gkac883)
Supplement: gkac883_Supplemental_File [file gkac883_supplemental_file.pdf]

Supplementary Materials

SIGNOR 3.0, the SIGnaling Network Open Resource 3.0: 2022 update.

Prisca Lo Surdo, Marta Iannuccelli, Silvia Contino, Luisa Castagnoli, Luana Licata, Gianni Cesareni *and Livia Perfetto*
*Corresponding authors

SIGNOR 3.0 score

The SIGNOR 3.0 significance score ranges from 0.1 and 1. We set 0.1 as the minimum score as 0 stands for no evidence of interaction. The significance scores of interactions between entities of classes *protein*, *proteinfamily* and *complex* is calculated by using a principal component regression (PCR) approach (1). This approach produces a model that predicts whether the annotated functional relationship between an entity pair is part of a molecular pathway annotated in our resource. The dataset of functional interactions which are part of SIGNOR molecular pathways is taken as the golden standard of functional interactions.

Briefly, we first created a matrix where each interacting pair is associated with the following features:

1) amount of evidence indicated as the number of PubMed IDs (PMIDs) associated to the pair, as annotated in SIGNOR;
2) A value that reflects whether the interaction has annotation direct yes/no. Specifically, yes=2, no=0, unknown= 1;
3) STRING 'coexpression' score, ranging from 0 to 1000;
4) STRING 'database' score, ranging from 0 to 1000;
5) STRING 'experimental' score, ranging from 0 to 1000;
6) STRING score 'textmining' score, ranging from 0 to 1000.

A score was also associated with interactions between proteins and protein families (PF) or complexes (CPX) by remapping STRING scores to interactions involving these entities according to the following criteria:

1) To calculate the interaction score between a protein X and a PF we obtained from STRING the individual interaction scores between the protein X and each PF -member and we associated the highest value to the protein X -PF interaction (Supplementary Figure 5A).
2) For interactions between a protein X and a CPX, for each feature, we retrieved from STRING the interaction scores between each complex subunit and protein X and we associated to the CPX-protein X interaction the mean of the individual subunit scores (Supplementary Figure 5B)
3) For PF-PF, CPX-CPX, PF-CPX and CPX-PF interactions, combinations of the aforementioned approaches were applied (Supplementary Figure 5A-B).

The PCR model was trained using the score matrix for protein-protein interactions having non-zero values for STRING scores.
We used the 'pls' package in R ('pcr' function, using cross validation as validation choice).

The regression coefficients to predict the dependent variable (probability of being part of an annotated pathway) were estimated on different numbers of principal components of the explanatory variables, ranging from 2 to 6.
We evaluated the performance achieved by using the different numbers of principal components, by visually checking the score distribution obtained with each scoring model for the full interactome (in blue in Supplementary Figure 5C-G) and for interactions annotated to pathways (in yellow in Supplementary Figure 5C-G).
As shown in Supplementary Figure 5C-G, by using three components (Supplementary Figure 5D) we obtained a model that maximizes the number of components used, still scores low the interactions for which we have poor supporting information, while scoring high interactions annotated to pathways. Thus, we eventually selected to use a model based on three principal components. The predicted score is then normalized between 0.1 and 1.
Scores for Interactions involving non-protein entities, which are considered in the SIGNOR network (*small molecules, phenotypes, stimuli, chemicals, fusion proteins, miRNA and antibodies)*, could not be assigned using the PCR model as many of the features considered for building the model were not available. For these interactions that represent a minority In the SIGNOR network we assigned an *ad hoc* score that reflects our confidence in the overall reliability of the supporting evidence. For Interactions involving

- chemicals and small molecules -> SIGNOR 3.0 significance score = 0.8
- Stimuli and phenotypes -> SIGNOR 3.0 significance score = 0.7
- Antibodies and miRNAs -> SIGNOR 3.0 significance score = 0.4
- Fusion proteins -> SIGNOR 3.0 significance score = 0.1

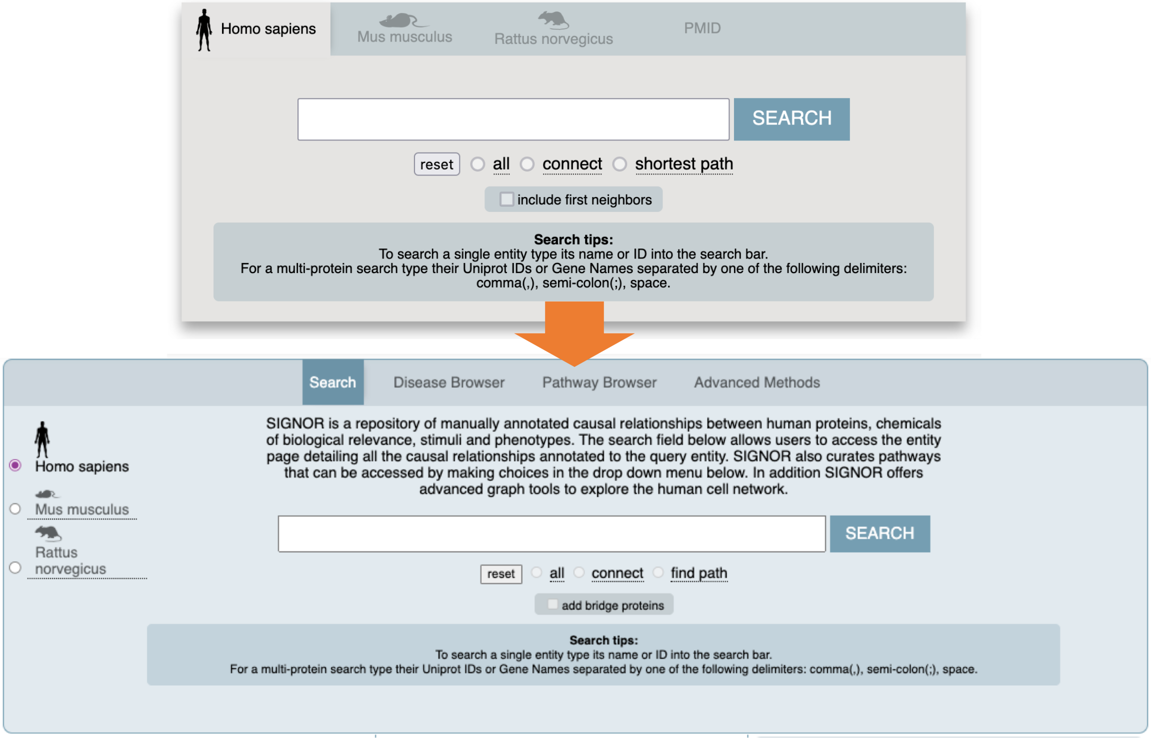

Supplementary Figure 1. SIGNOR search box. Comparison between SIGNOR 2.0 (top) and SIGNOR 3.0(bottom) search boxes.

A

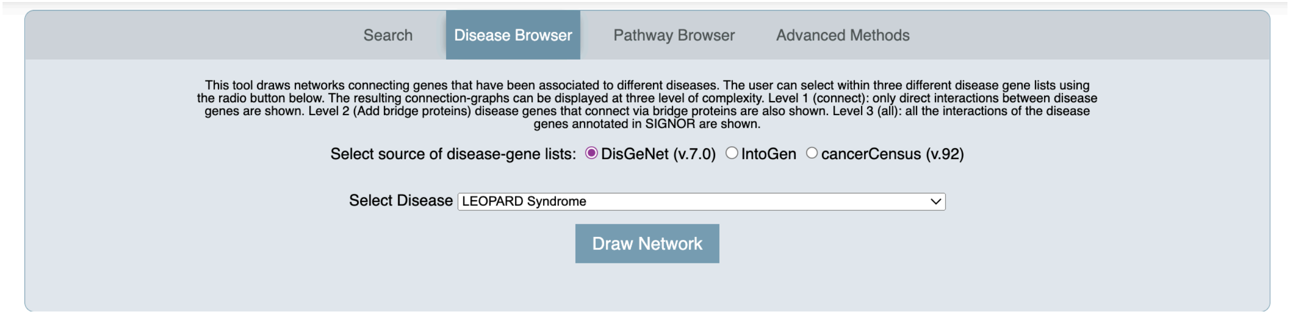

B

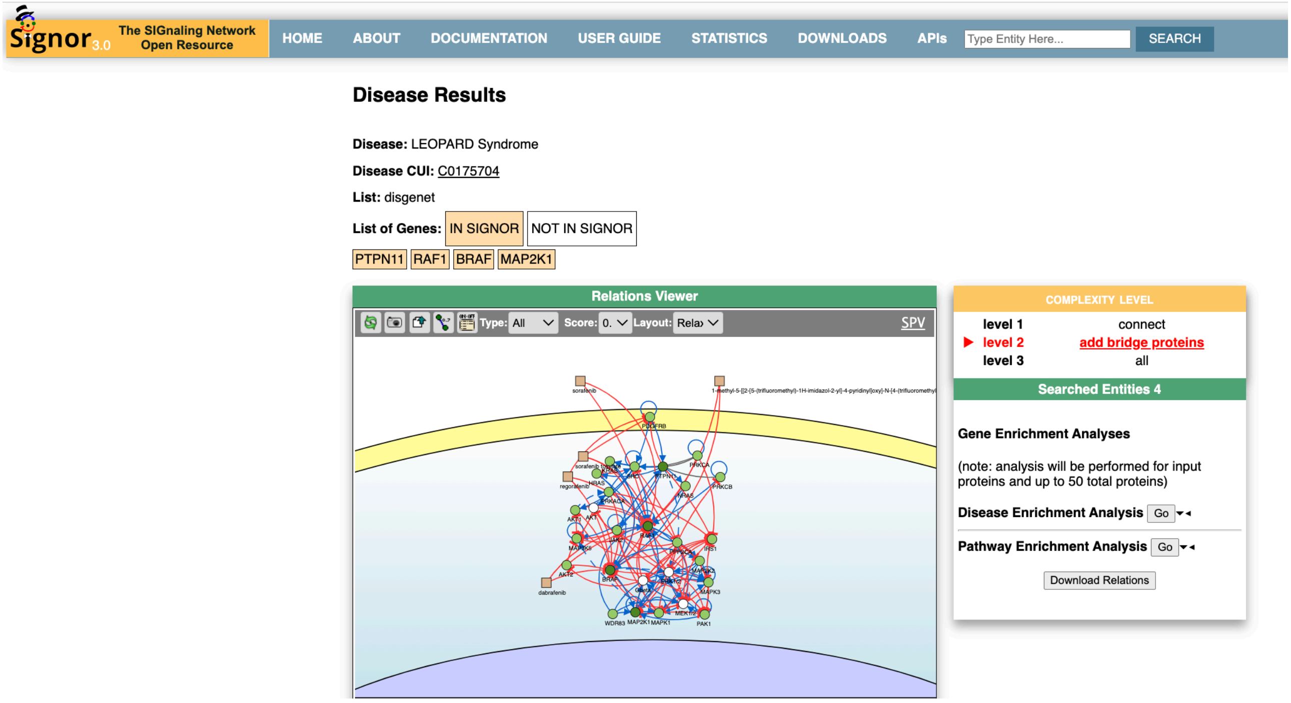

Supplementary Figure 2. The Disease Brower. (A) The *Disease Browser* tab allows the user to select a source of disease-gene lists annotated in *DisGeNET, IntOGen* or *Cancer Gene Census* resources (2–4). **(B)** Example of a Disease result page (LEOPARD syndrome). The results page reports: a summary of the disease and the list of genes associated with it, as provided in the source database (top); the graph viewer recapitulating the interactions occurring between these genes (middle); the complexity level box (top-right) that allows the user to display the interactions using three search methods: *connect*, *add bridge proteins* and *all*, the default option is add bridge proteins. Further details about the search methods are provided by Lo Surdo et al (5); the enrichment tool (bottom-right) that allows the user to perform a gene set enrichment analysis on KEGG-defined pathways or on other disease annotations.

A

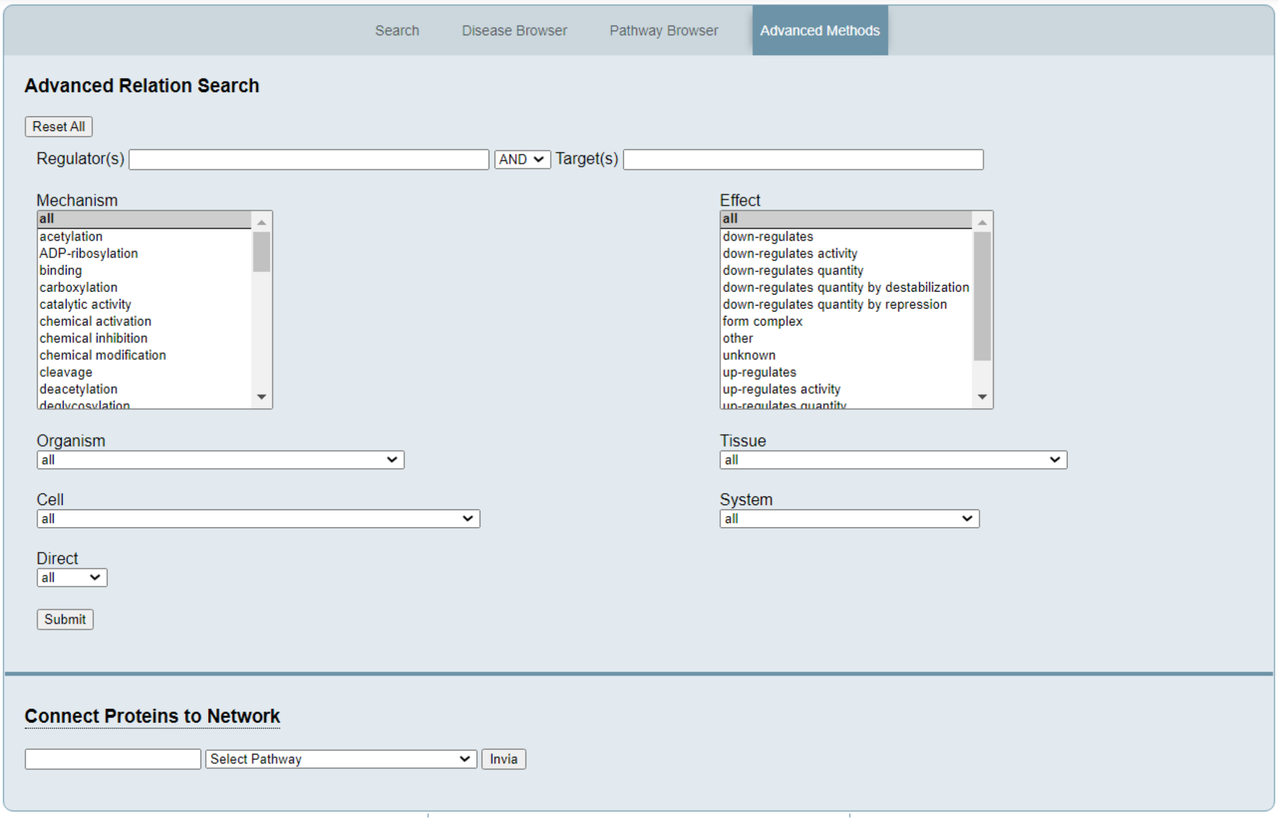

B

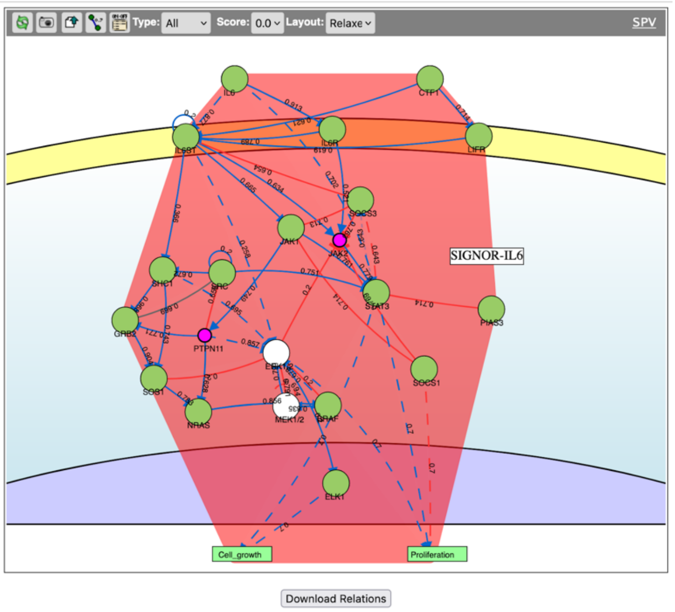

Supplementary Figure 3. The Advanced methods tab. (A) The top section allows the user to perform a more refined search. Among the options it is possible to: specify a list of regulators and/or targets; mechanisms annotated in the interactions; effects annotated in the interactions; organism, tissue, cell or system as defined by the BRENDA ontology (6) annotated in the interactions; specify whether resulting interactions should be filtered as direct or indirect.
(B) The bottom section includes a tool that allows the user to search for a connection (of no more than two steps) between a list of input proteins and a SIGNOR pathway selected from the dropdown menu.

A

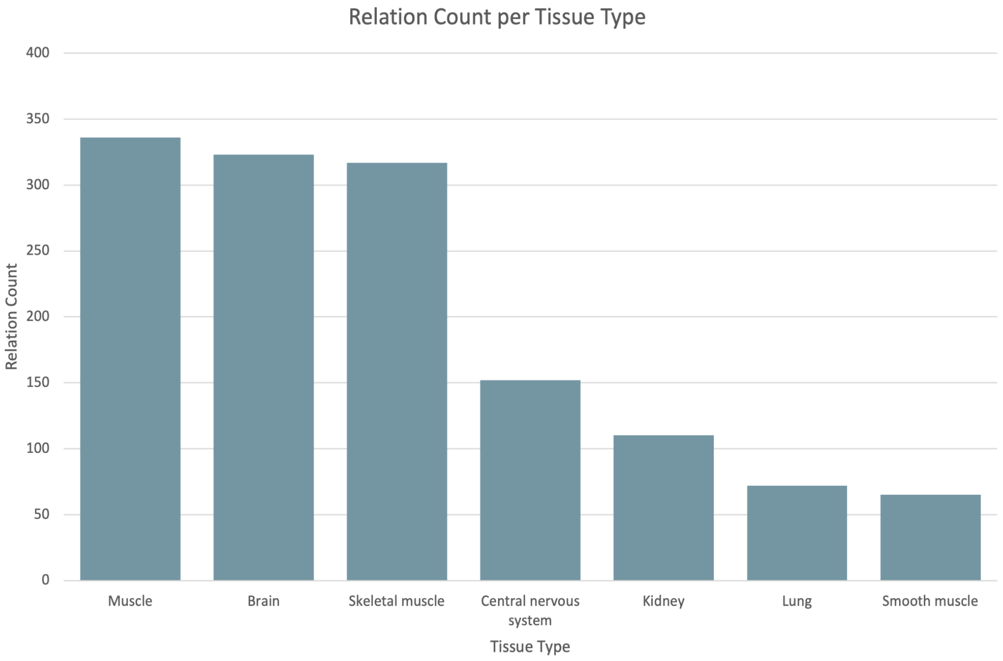

B

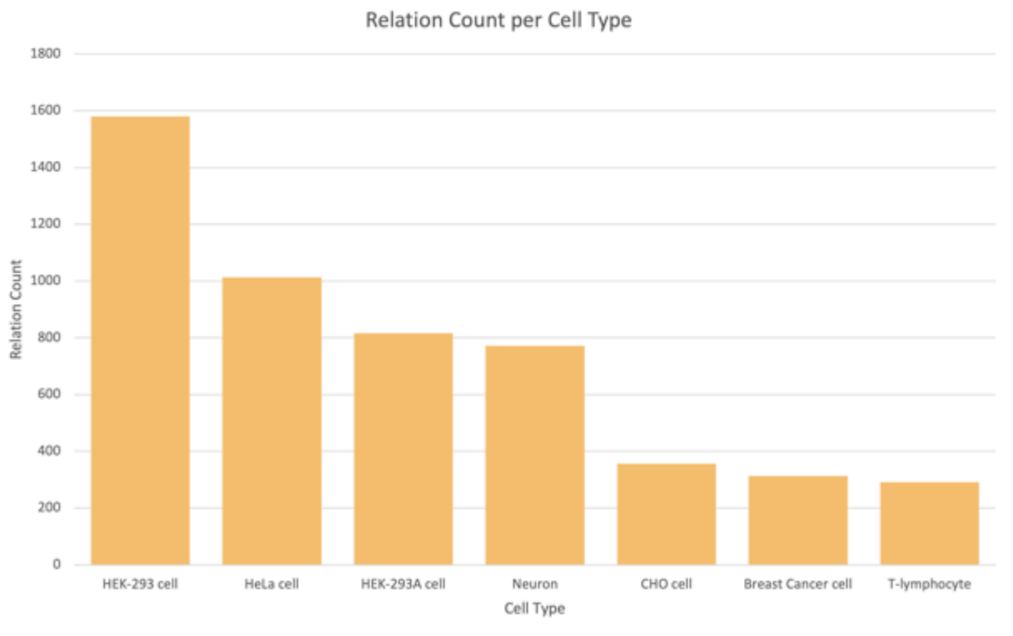

Supplementary Figure 4. Most represented biological systems associated with SIGNOR 3.0 interactions. Interaction Count associated with the top 7 most represented tissue **(A)** or cell types **(B)**, as defined by the Brenda ontology (6).

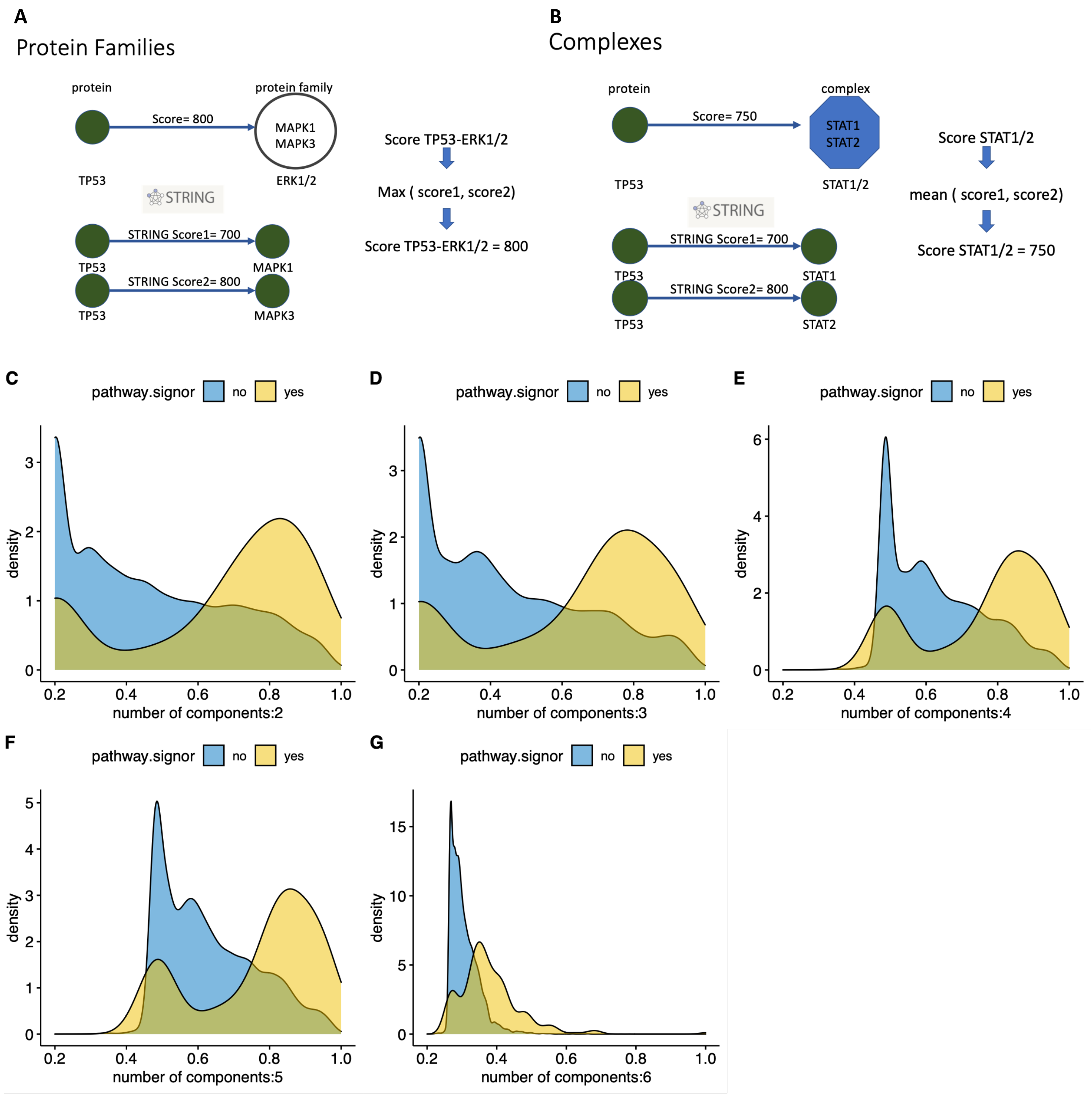

Supplementary Figure 5. SIGNOR 3.0 score. (A) Protein-protein family interaction score. For interactions between a protein (e.g. TP53) and a protein family (e.g. ERK1/2) we obtained from STRING the individual interaction scores between the protein and each family member (MAPK1 and MAPK3): score 1 and score2. We then associated the interaction between the protein family entity and the protein with a score that is the maximum of the individual scores (Max(score1, score2)). **(B)** Protein-complex interaction score. For interactions between a protein (e.g. TP53) and a protein complex (e.g. STAT1/2) we obtained from STRING the interaction scores between the protein and each complex member (e.g. STAT1 and STAT2): score 1 and score2. We then associated the interaction between the complex entity and the protein with a score that is the average of the individual scores (Av(score1, score2)). **(C-G)** Score distribution of interactions involving protein, protein complexes and protein families by number of components. In yellow interactions annotated to a pathway, in blue the rest of the interactome

References

1. Mevik,B.-H. and Wehrens,R. (2007) The pls Package: Principal Component and Partial Least Squares Regression in R. *Journal of Statistical Software*, **18**, 1–23.

2. Piñero,J., Ramírez-Anguita,J.M., Saüch-Pitarch,J., Ronzano,F., Centeno,E., Sanz,F. and Furlong,L.I. (2020) The DisGeNET knowledge platform for disease genomics: 2019 update. *Nucleic Acids Res*, **48**, D845–D855.

3. Gonzalez-Perez,A., Perez-Llamas,C., Deu-Pons,J., Tamborero,D., Schroeder,M.P., Jene-Sanz,A., Santos,A. and Lopez-Bigas,N. (2013) IntOGen-mutations identifies cancer drivers across tumor types. *Nat Methods*, **10**, 1081–1082.

4. Sondka,Z., Bamford,S., Cole,C.G., Ward,S.A., Dunham,I. and Forbes,S.A. (2018) The COSMIC Cancer Gene Census: describing genetic dysfunction across all human cancers. *Nat Rev Cancer*, **18**, 696–705.

5. Lo Surdo,P., Calderone,A., Iannuccelli,M., Licata,L., Peluso,D., Castagnoli,L., Cesareni,G. and Perfetto,L. (2018) DISNOR: a disease network open resource. *Nucleic Acids Res*, **46**, D527–D534.

6. Chang,A., Schomburg,I., Placzek,S., Jeske,L., Ulbrich,M., Xiao,M., Sensen,C.W. and Schomburg,D. (2015) BRENDA in 2015: exciting developments in its 25th year of existence. *Nucleic Acids Res*, **43**, D439-446.
